# Relationship between neutropenia caused by nanoliposomal irinotecan/fluorouracil/leucovorin and treatment outcomes in the NAPOLEON-2 study (NN-2301)

**DOI:** 10.1038/s41598-025-88005-4

**Published:** 2025-01-27

**Authors:** Tomonori Araki, Yuki Sonoda, Mototsugu Shimokawa, Taiga Otsuka, Kohei Hayashi, Takuya Honda, Kazuhiko Nakao, Taro Shibuki, Junichi Nakazawa, Shiho Arima, Keisuke Miwa, Yoshinobu Okabe, Futa Koga, Yujiro Ueda, Yoshihito Kubotsu, Hozumi Shimokawa, Shigeyuki Takeshita, Azusa Komori, Kazuo Nishikawa, Satoshi Otsu, Ayumu Hosokawa, Hisanobu Oda, Tatsunori Sakai, Shuji Arita, Machiko Kawahira, Hiroki Taguchi, Kengo Tsuneyoshi, Yasunori Kawaguchi, Toshihiro Fujita, Takahiro Sakae, Tsuyoshi Shirakawa, Toshihiko Mizuta, Kenji Mitsugi

**Affiliations:** 1 Department of Gastroenterology and Hepatology, Graduate School of Biomedical Sciences, Nagasaki University, Nagasaki, Japan; 2 Clinical Research Institute, National Kyushu Cancer Center, Fukuoka, Japan; 3 Department of Biostatistics, Yamaguchi University Graduate School of Medicine, Yamaguchi, Japan; 4 Department of Internal Medicine, Minato Medical Clinic, Fukuoka, Japan; 5 Department for the Promotion of Drug and Diagnostic Development, Division of Drug and Diagnostic Development Promotion, Translational Research Support Office, National Cancer Center Hospital East, Chiba, Japan; 6 Department of Hepatobiliary and Pancreatic Oncology, National Cancer Center Hospital East, Chiba, Japan; 7 Department of Medical Oncology, Kagoshima City Hospital, Kagoshima, Japan; 8 Digestive and Lifestyle Diseases, Kagoshima University Graduate School of Medical and Dental Sciences, Kagoshima, Japan; 9 Multidisciplinary Treatment Cancer Center, Kurume University Hospital, Fukuoka, Japan; 10 Division of Gastroenterology, Department of Medicine, Kurume University School of Medicine, Fukuoka, Japan; 11 Department of Hepatobiliary and Pancreatology, Saga Medical Center Koseikan, Saga, Japan; 12 Department of Hematology and Oncology, Japanese Red Cross Kumamoto Hospital, Kumamoto, Japan; 13 Department of Internal Medicine, Karatsu Red Cross Hospital, Saga, Japan; 14 Department of Hematology Oncology, Community Healthcare Organization Kyushu Hospital, Fukuoka, Japan; 15 Department of Gastroenterology, Japanese Red Cross Nagasaki Genbaku Hospital, Nagasaki, Japan; 16 Department of Medical Oncology and Hematology, Oita University Faculty of Medicine, Oita, Japan; 17 Department of Gastrointestinal Medical Oncology, National Hospital Organization Shikoku Cancer Center, Ehime, Japan; 18 Department of Clinical Oncology, University of Miyazaki Hospital, Miyazaki, Japan; 19 Division of Integrative Medical Oncology, Saiseikai Kumamoto Hospital, Kumamoto, Japan; 20 Department of Medical Oncology, NHO Kumamoto Medical Center, Kumamoto, Japan; 21 Department of Chemotherapy, Miyazaki Prefectural Miyazaki Hospital, Miyazaki, Japan; 22 Department of Gastroenterology, Kagoshima Kouseiren Hospital, Kagoshima, Japan; 23 Department of Gastroenterology, Izumi General Medical Center, Kagoshima, Japan; 24 Department of Gastroenterology, Kagoshima City Hospital, Kagoshima, Japan; 25 Department of Gastroenterology, Asakura Medical Association Hospital, Fukuoka, Japan; 26 Department of Gastroenterology, Saiseikai Sendai Hospital, Kagoshima, Japan; 27 Researcher of Clinical Hematology Oncology Treatment Study Group, 1-14-6 Muromi-gaoka, Nishi-ku, Fukuoka-shi, Fukuoka 819-0030 Japan; 28 Director of Medical Checkup Center, Eikoh Hospital, 3-8-15 Befu-nishi, Shime-machi, Kasuya- gun, Fukuoka, 811-2232 Japan; 29 Department of Internal Medicine, Fujikawa Hospital, Saga, Japan; 30 Department of Medical Oncology, Sasebo Kyosai Hospital, Sasebo, Japan

**Keywords:** Cancer, Gastrointestinal cancer

## Abstract

The relationship between nanoliposomal irinotecan/fluorouracil/leucovorin (NFF) treatment outcomes and neutropenia in patients with pancreatic cancer has not been thoroughly examined. Thus, we conducted a retrospective analysis of data from patients with pancreatic cancer who were treated with NFF to investigate this relationship. Neutropenia was assessed according to the Common Terminology Criteria for Adverse Events across three cutoffs: A (grade 0 versus grade 1–4), B (grades 0–1 versus 2–4), and C (grades 0–2 versus 3–4). The primary endpoint was overall survival (OS), and the secondary endpoints were overall response rate, progression-free survival (PFS), and relative dose intensity. Of the 161 patients, 93, 8, 22, 30, and 8 patients had neutropenia of grades 0, 1, 2, 3, and 4, respectively. The overall response rates differed significantly at cutoff C (*p* = 0.02), with the odds ratio for cutoff C being the highest, followed by cutoffs B and A. Significant differences in OS were observed at cutoffs A (hazard ratio [HR], 0.65; 95% confidence interval [CI], 0.44–0.94; *p* = 0.02) and B (HR, 0.63; 95% CI, 0.43–0.92, *p* = 0.02). Similarly, PFS showed significant differences at cutoffs A and B (*p* < 0.01). NFF-induced neutropenia can be a useful prognostic factor for patients with pancreatic cancer.

**Supplementary Information:**

The online version contains supplementary material available at 10.1038/s41598-025-88005-4.

## Introduction

Pancreatic cancer is one of the deadliest malignancies worldwide and is the third leading cause of cancer-related death^1^. Based on the results of the ACCORD11 and MPACT trials^2,3^, 5-fluorouracil (5-FU), leucovorin (LV), irinotecan (IRI) plus oxaliplatin, and gemcitabine plus nab-paclitaxel were approved as first-line chemotherapeutic agents for metastatic pancreatic cancer. In addition, 5-FU, LV, and nanoliposomal IRI (nal-IRI) were approved as second-line treatments based on the findings of the NAPOLI-1 study^4,5^. Moreover, nal-IRI was shown to be effective when added to 5-FU and LV in Japanese patients and was subsequently approved for use in Japan^6^. Nal-IRI is reported to have increased permeability and retention in cancer patients, allowing it to accumulate more effectively in tumor tissues. Additionally, nal-IRI has a longer half-life than non-liposomal IRI. However, the overall toxicity of nal-IRI is not significantly different from that of non-liposomal IRI^7^. Despite numerous studies investigating potential biomarkers for nal-IRI^8–12^, evidence regarding its toxicity and efficacy remains unclear. Specifically, the relationship between the extent of neutropenia, a characteristic side effect of nal-IRI, and treatment outcomes has not been examined.

The relationship between neutropenia and the efficacy of TAS-102 in colorectal cancer is well established^13^. In pooled analyses of prospective studies, this relationship has also been reported in lung cancer and across various solid tumors^14–16^. Hematologic toxicity intensity is considered an indicator of cytotoxic activity, with severe myelosuppression viewed as a marker of increased blood levels of anticancer drugs^14,17^. It is important to investigate the relationship between neutropenia and the efficacy of nal-IRI plus 5-FU/LV (NFF) in pancreatic cancer treatment because nal-IRI often causes neutropenia^5,18^.

In this study, we explored the potential of NFF-induced neutropenia as a useful prognostic factor for pancreatic cancer treatment. By elucidating the relationship between neutropenia and treatment outcomes, our aim is to contribute to treatment decision-making and improve the overall prognosis for patients with pancreatic cancer.

## Results

### Patients

**Fig. 1:**
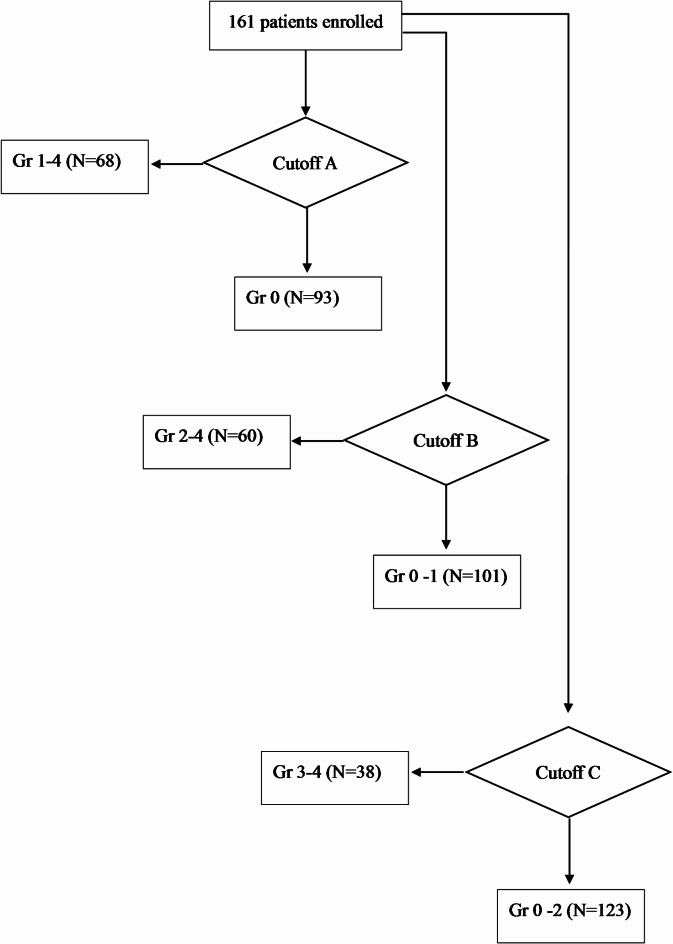
Flow diagram. Of the 161 patients included, Gr 0, 1, 2, 3, and 4 neutropenia was observed in 93, 8, 22, 30, and 8 patients, respectively. The patients were divided into different groups based on the cutoff neutrophil counts. At cutoff A, 93 patients were in the Gr 0 group, and 68 patients were in the Gr 1–4 group. At cutoff B, 101 patients were in the Gr 0–1 group and 60 patients were in the Gr 2–4 group. At cutoff C, 123 patients were in the Gr 0–2 group and 38 patients were in the Gr 3–4 group.

A total of 161 pancreatic cancer patients were included in this study. The median follow up was 7.5 months (range, 0.3–20.7 months). Of the 161 patients, 93, 8, 22, 30, and 8 patients had Gr 0, 1, 2, 3, and 4 neutropenia, respectively. Patients were divided into different groups based on neutrophil counts (Fig. 1). At cutoff A, there were 93 patients in the Gr 0 group and 68 patients in the Gr 1–4 group. White blood cell (WBC) counts, neutrophil counts, lymphocyte counts, C-reactive protein (CRP) levels, and the 5-FU relative dose intensity (RDI) differed significantly between the groups. At cutoff B, there were 101 patients in the Gr 0–1 group and 60 patients in the Gr 2–4 group, with significant differences also noted in these parameters. At cutoff C, there were 123 patients in the Gr 0–2 group and 38 patients in the Gr 3–4 group. Significant differences were found in WBC counts, neutrophil counts, and the 5-FU RDI (Table 1).

**Table 1:** Patient characteristics according to neutropenia grade cutoffs and tumor responses.

	Cutoff A			Cutoff B			Cutoff C		
	Gr 0 (*N* = 93)	Gr 1–4 (*N* = 68)	p-value	Gr 0–1 (*N* = 101)	Gr 2–4 (*N* = 60)	p-value	Gr 0–2 (*N* = 123)	Gr 3–4 (*N* = 38)	p-value
Age, years	67 (38–85)	67.5 (47–79)		67 (38–85)	67.5 (47–79)		68 (38–85)	66.5 (47–79)	
Sex									
Female	37 (40%)	36 (53%)	0.11	41 (41%)	32 (53%)	0.14	55 (45%)	18 (47%)	0.85
ECOG Performance status									
0	46 (49%)	28 (41%)	0.29	47 (47%)	27 (45%)	0.87	59 (48%)	15 (39%)	0.67
1	43 (46%)	33 (49%)		48 (48%)	28 (47%)		56 (46%)	20 (53%)	
≥ 2	4 (4%)	7 (10%)		6 (6%)	5 (8%)		8 (7%)	3 (8%)	
Malignant history									
Yes	16 (17%)	9 (13%)	0.52	19 (19%)	6 (10%)	0.18	22 (18%)	3 (8%)	0.20
Malignant family history									
Yes	35 (38%)	28 (41%)	0.74	38 (38%)	25 (42%)	0.62	49 (40%)	14 (37%)	0.85
Previous tumor resection									
Yes	22 (24%)	18 (26%)	0.71	24 (24%)	16 (27%)	0.71	28 (23%)	12 (32%)	0.29
Pancreatic tumor location									
Head	36 (39%)	31 (46%)	0.18	41 (41%)	26 (43%)	0.27	52 (42%)	15 (39%)	0.07
Body	32 (34%)	27 (40%)		34 (34%)	25 (42%)		40 (33%)	19 (50%)	
Tail	25 (27%)	10 (15%)		26 (26%)	9 (15%)		31 (25%)	4 (11%)	
Histologic subtype									
Adenocarcinoma	87 (94%)	61 (90%)	0.11	95 (94%)	53 (88%)	0.06	114 (93%)	34 (89%)	0.25
Others	5 (5%)	2 (3%)		5 (5%)	2 (3%)		6 (5%)	1 (3%)	
Unknown	1 (1%)	5 (7%)		1 (1%)	5 (8%)		3 (2%)	3 (8%)	
Stage									
Locally advanced	10 (11%)	9 (13%)	0.77	11 (11%)	8 (13%)	0.76	16 (13%)	3 (8%)	0.49
Metastatic	61 (66%)	41 (60%)		66 (65%)	36 (60%)		79 (64%)	23 (61%)	
Recurrence	22 (24%)	18 (26%)		24 (24%)	16 (27%)		28 (23%)	12 (32%)	
Site of metastatic disease									
Liver	54 (58%)	35 (51%)	0.43	57 (56%)	32 (53%)	0.74	70 (57%)	19 (50%)	0.46
Lung	17 (18%)	11 (16%)	0.83	19 (19%)	9 (15%)	0.67	20 (16%)	8 (21%)	0.47
Peritoneum	26 (28%)	18 (26%)	0.86	28 (28%)	16 (27%)	1.00	34 (28%)	10 (26%)	1.00
Ascites									
Abdominal	2 (2%)	5 (7%)	0.27	4 (4%)	3 (5%)	0.77	6 (5%)	1 (3%)	1.00
Pelvic	11 (12%)	6 (9%)		12 (12%)	5 (8%)		13 (11%)	4 (11%)	
no	80 (86%)	57 (84%)		85 (84%)	52 (87%)		104 (84%)	33 (86%)	
No. of previous chemotherapy regimens									
1	64 (69%)	40 (59%)	0.43	69 (68%)	35 (58%)	0.35	82 (67%)	22 (58%)	0.33
2	21 (23%)	20 (29%)		24 (24%)	17 (28%)		31 (25%)	10 (26%)	
≥ 3	8 (9%)	8 (12%)		8 (8%)	8 (13%)		10 (8%)	6 (16%)	
Previous treatment									
FOLFIRINOX1st/2nd/3rd line	6/3/2	1/4/2		6/4/2	1/3/2		6/4/3	1/3/1	
GEM + nab-PTX1st/2nd/3rd line	74/17/3	56/12/2		81/18/3	49/11/2		98/23/3	32/6/2	
nal-IRI course									
Median (range)	5 (1–38)	6 (1–36)		5 (1–38)	6.5 (2–36)		5 (1–38)	6 (2–36)	
5-FU course									
Median (range)	5 (1–38)	6 (1–36)		5 (1–38)	6.5 (2–36)		5 (1–38)	6 (2–36)	
UGT1A1 *6/*28									
Wild	53 (57%)	27 (40%)	0.05	57 (56%)	23 (38%)	0.07	67 (54%)	13 (34%)	0.08
Single hetero	29 (31%)	35 (51%)		33 (33%)	31 (52%)		43 (35%)	21 (55%)	
Double hetero	1 (1%)	2 (3%)		1 (1%)	2 (3%)		2 (2%)	1 (3%)	
Homozygous	3 (3%)	2 (3%)		3 (3%)	2 (3%)		3 (2%)	2 (5%)	
Unknown	7 (8%)	2 (3%)		7 (7%)	2 (3%)		8 (7%)	1 (3%)	
WBC base	6800 (3000–22770)	5050 (2850–27100)	< 0.05	6800 (3000–22770)	4970 (2850–27100)	< 0.05	6500 (2850–27100)	5300 (3000–17000)	< 0.05
WBC ≥ 11,000	8 (9%)	2 (3%)	0.19	8 (8%)	2 (3%)	0.32	9 (7%)	1 (3%)	0.45
Ne base	4520 (1241–19900)	3345 (1430–23171)	< 0.05	4485 (1241–19900)	3250 (1430–23171)	< 0.05	4100 (1241–23171)	3372 (1750–13900)	< 0.05
Ly base	1326 (408–3430)	1100 (440–3184)	< 0.05	1334.0 (408–3430)	1099.5 (440–3101)	< 0.05	1239.0 (408–3430)	1079.5 (440–2484)	0.14
Hb base	10.6 (7.3–14.2)	10.65 (7.1–13.7)	0.28	10.6 (7.3–14.2)	10.7(7.1–13.4)	0.70	10.6 (7.1–14.2)	10.7 (7.2–13.4)	0.81
Plt base	26.0 (6.7–68.5)	22.7(8.9–53.6)	0.07	26.0 (6.7–68.5)	22.3(8.9–53.6)	0.07	25.40 (6.7–68.5)	23.25 (8.9–52.3)	0.62
Alb base	3.6 (2.1–4.8)	3.7 (1.9–4.7)	0.37	3.6 (2.1–4.8)	3.7 (1.9–4.7)	0.21	3.65 (1.9–4.8)	3.70 (2.5–4.7)	0.68
AST base	23.9 (11–232)	24.5 (13–112)	0.18	24 (11–232)	24 (13–112)	0.44	24.0 (11–232)	24.5 (15–60)	0.36
ALT base	19 (5–128)	21 (7–127)	0.15	19 (5–128)	21 (7–127)	0.34	20.0 (5–128)	21.5 (9–127)	0.27
LDH base	201 (113–798)	209 (74–911)	0.70	201 (113–798)	209 (74–911)	0.36	204.5 (113–798)	204.0 (74–911)	0.69
Cr base	0.64 (0.39–1.31)	0.60 (0.40–1.66)	0.11	0.64 (0.39–1.66)	0.60 (0.40–1.08)	0.25	0.63 (0.39–1.66)	0.63 (0.40–0.97)	0.79
CRP base	0.42 (0.01–16.91)	0.21 (0.01–21.93)	< 0.05	0.440 (0.01–16.91)	0.185 (0.01–21.93)	< 0.05	0.34 (0.01–21.93)	0.175 (0.01–4.45)	0.05
CEA base	11.7 (1.7–1599)	8.8 (1.0–6342)	0.15	11.05 (1.7–6342)	8.80 (1.0–192)	0.15	10.65 (1.3–6342.0)	8.60 (1.0–93.6)	0.15
CA19_9 base	1252.0 (0.6–543522)	742.6 (0.6–61506)	0.30	1320.0 (0.6–543522)	741.2 (0.6–61506)	0.28	1320.0 (0.6–543522)	693.2 (1.3–61506)	0.42
5-FU RDI	97.10 (55.4–108.3)	84.55 (40.8–105.9)	< 0.05	96.30 (40.8–108.3)	84.55 (50.4–103.6)	< 0.05	95.1 (40.8–108.3)	83.1 (50.4–103.6)	< 0.05
nal-IRI RDI	83.1 (56.7–105.7)	81.1 (53.0–102.8)	0.10	83.1 (56.7–105.7)	81.1 (53.0–102.8)	0.09	83.20 (56.7–105.7)	80.55 (53.0–102.8)	0.14
Response	2 (1%)	6 (4%)		2 (1%)	6 (4%)		3 (2%)	5 (3%)	

At cutoff A, there were 71 overall survival (OS) events in the Gr 0 group and 46 in the Gr 1–4 group. At cutoff B, there were 77 OS events in the Gr 0–1 group and 40 in the Gr 2–4 group. Finally, at cutoff C, there were 91 OS events in the Gr 0–2 group and 26 in the Gr 3–4 group. The number of progression-free survival (PFS) events was 90 in the Gr 0 group and 60 in the Gr 1–4 group at cutoff A, 97 in the Gr 0–1 group and 53 in the Gr 2–4 group at cutoff B, and 115 in the Gr 0–2 group and 35 in the Gr 3–4 group at cutoff C. In total, the number of PFS events was 150. Overall, 11 patients (6.8%) continued NFF treatment, whereas 150 patients (93.2%) discontinued treatment due to disease progression (*n* = 134, 89.3%), adverse events (AEs) (*n* = 12, 8.0%), patient refusal (*n* = 2, 1.3%), and other causes (*n* = 2, 1.3%).

### Tumor response

The relationship between each cutoff and tumor response was as follows: The odds ratios for tumor response were 4.40 (95% confidence interval [CI]^19^ 0.86–22.53; *p* = 0.07) at cutoff A and 5.50 (95% CI 1.07–28.19; *p* = 0.05) at cutoff B. However, there was a significant difference at cutoff C (odd ratio 6.06; 95% CI 1.38–26.69; *p* = 0.02). The magnitude of the odds ratios exhibited a consistent descending pattern, with cutoff C demonstrating the highest value, followed by B, and finally A with the lowest. The logistic regression analysis further confirmed these findings. Significant differences were found at cutoff A (*p* = 0.04; odds ratio 3.54; 95% CI, 0.64–19.50), cutoff B (*p* = 0.02; odds ratio 5.74; 95% CI, 1.00-32.50), and cutoff C (*p* = 0.01; odds ratio 5.87; 95% CI 1.20–28.80) (Table 1).

### OS and PFS

**Fig. 2:**
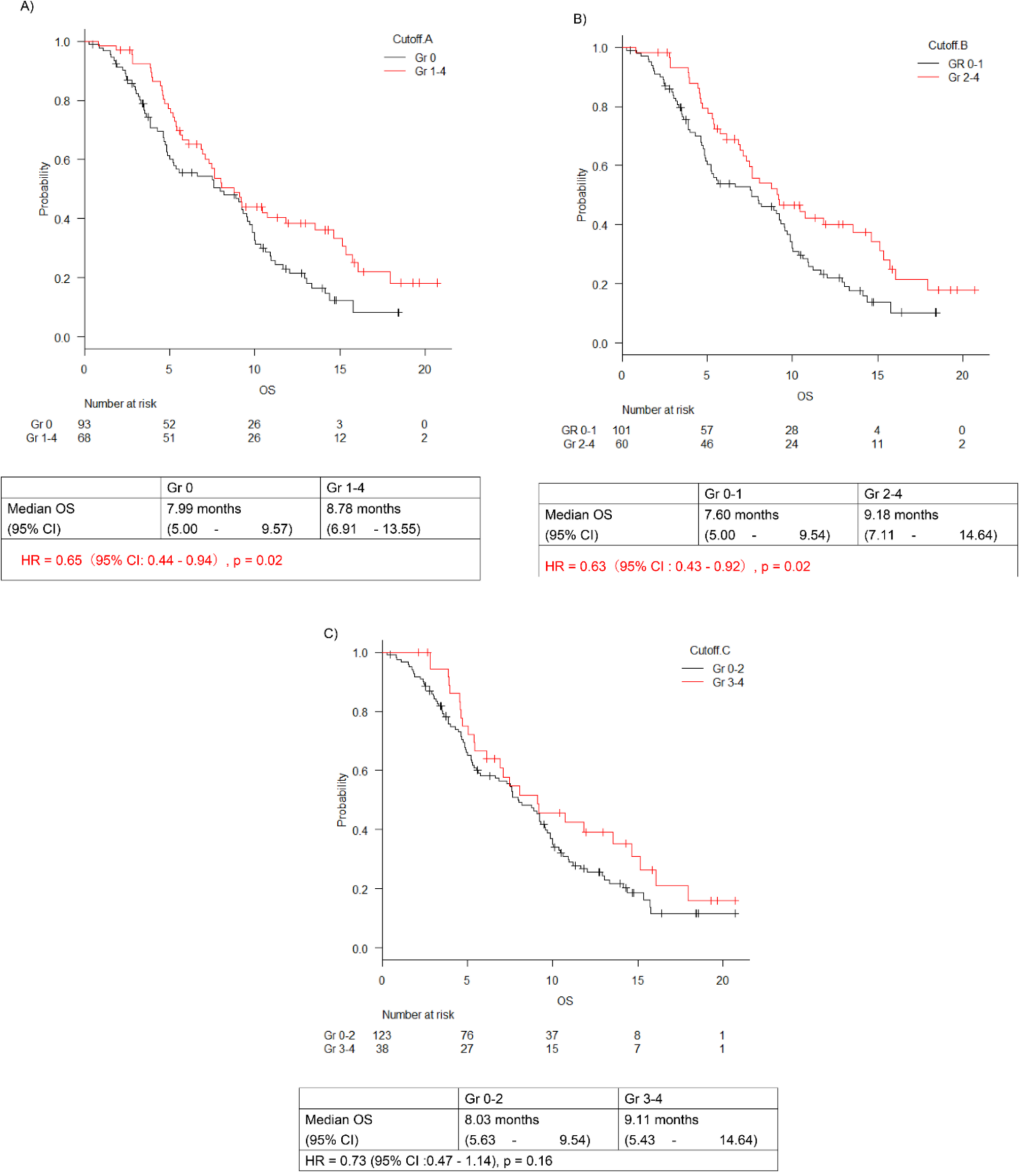
Overall survival analysis. Overall survival analysis of patients with pancreatic cancer based on different neutropenia grade cutoffs (**A**: cutoff A, **B**: cutoff B, and **C**: cutoff C). The graph shows the survival curves and corresponding hazard ratios for each cutoff group. Cutoffs A and B show significant differences. However, cutoff C shows a trend toward improved survival, although the difference is not statistically significant.

OS analysis showed that the group with higher neutropenic grades at cutoffs A and B had significantly better OS, whereas the group with higher neutropenic grades at cutoff C showed a trend towards better OS, although the difference was not statistically significant (Fig. 2A-C). OS in the Gr 1–4 group was significantly better than in the Gr 0 group at cutoff A (HR 0.646; 95% CI 0.44–0.94; *p* = 0.02) (Fig. 2A). Similarly, OS in the Gr 2–4 group was significantly better than the Gr 0–1 group at cutoff B (HR 0.63; 95% CI 0.43–0.92; *p* = 0.02) (Fig. 2B). OS in the Gr 3–4 group was significantly better than that in the Gr 0–2 group at cutoff C (HR 0.73; 95% CI 0.47–1.14; *p* = 0.16) (Fig. 2C). Multivariate Cox regression analyses showed significant differences, with adjusted HRs of 0.65 (95% CI 0.43–0.97) at cutoff A and 0.66 (95% CI 0.44-1.00) at cutoff B. In contrast, no significant difference was observed at cutoff C with a HR of 0.77 (95% CI 0.48–1.23) (Table 2).

**Table 2:** Univariate and multivariate cox regression analyses.

		Univariate			Multivariate (Cutoff A)			Multivariate (Cutoff B)			Multivariate (Cutoff C)		
		HR	95% CI	p-value	HR	95% CI	p-value	HR	95% CI	p-value	HR	95% CI	p-value
Age	≥ 75	0.68	0.41–1.14	0.15	0.64	0.37–1.11	0.11	0.65	0.38–1.12	0.12	0.65	0.38–1.11	0.11
PS	≥ 1	1.38	0.95–1.99	0.09	1.39	0.94–2.04	0.10	1.37	0.93–2.02	0.11	1.38	0.94–2.05	0.11
No. of previous chemotherapy regimens	≥ 2	0.92	0.63–1.34	0.68	1.25	0.82–1.91	0.31	1.23	0.81–1.88	0.34	1.16	0.77–1.75	0.49
Ascites	Yes	2.26	1.38–3.70	< 0.01	2.39	1.40–4.08	< 0.01	2.29	1.34–3.90	< 0.01	2.29	1.33–3.93	< 0.01
Neutropenia	Gr 1–4 (Cutoff A)	0.65	0.44–0.94	0.02	0.65	0.43–0.97	0.03						
	Gr 2–4 (Cutoff B)	0.63	0.43–0.92	0.02				0.66	0.44-1.00	< 0.05			
	Gr 3–4 (Cutoff C)	0.73	0.47–1.14	0.16							0.77	0.48–1.23	0.28
CA19-9	≥ 1000	1.35	0.94–1.96	0.11	1.27	0.86–1.87	0.24	1.26	0.85–1.86	0.25	1.22	0.83–1.80	0.31
CRP	≥ 1	2.49	1.68–3.70	< 0.01	2.24	1.46–3.45	< 0.01	2.25	1.46–3.46	< 0.01	2.23	1.45–3.43	< 0.01
Stage	Metastatic, Recurrence	0.64	0.38–1.10	0.11	0.78	0.44–1.37	0.38	0.76	0.44–1.34	0.34	0.73	0.42–1.27	0.26

Age, carbohydrate antigen 19 − 9 (CA19-9), and CRP were included as continuous variables, alongside gender as a categorical variable, for each cutoff point. A sensitivity analysis using cox regression was performed to assess the robustness of these findings (Supplementary Table 1). However, CA19-9 was logarithmically transformed due to its wide range of values. Except for CRP, no significant differences were observed, although the group with higher neutropenia tended to show better OS across each cutoff, although the difference did not reach statistical significance. The number of missing values was two for CRP and four for CA19-9, while there were no missing values for age, PS, ascites, or the number of previous chemotherapy regimens. The number of previous chemotherapy regimens refers to the number of chemotherapy regimens administered before initiating NFF, which did not include preoperative and postoperative adjuvant therapy.

**Fig. 3:**
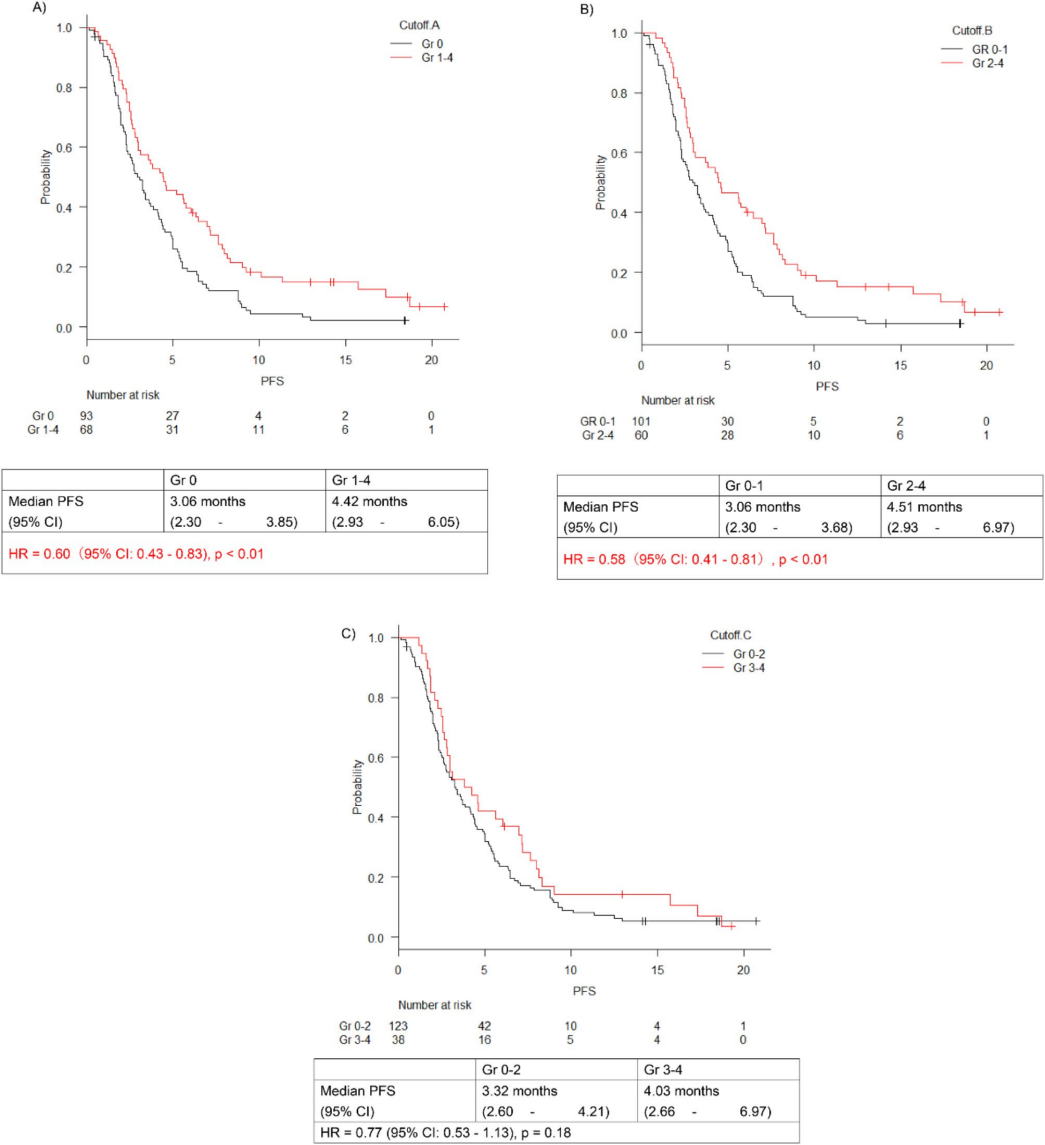
Progression-free survival analysis. Progression-free survival analysis of patients with pancreatic cancer using different neutropenia grade cutoffs (**A**: cutoff A, **B**: cutoff B, and **C**: cutoff C). The graph displays the survival curves and corresponding hazard ratios for each cutoff group. Cutoffs A and B show significant differences. Cutoff C also shows a trend toward improved survival, although the difference is not statistically significant.

PFS analysis similarly showed that the group with higher neutropenic grades at cutoffs A and B exhibited significantly better PFS, and the group with higher neutropenic grades at cutoff C tended to have better PFS, although the difference was not significant (Fig. 3A-C). PFS in the Gr 1–4 group was significantly better than the Gr 0 group at cutoff A (HR, 0.60; 95% CI 0.43–0.83; *p* < 0.01) (Fig. 3A). PFS in the Gr 2–4 group showed better than the Gr 0–1 group at cutoff B (HR 0.578; 95% CI 0.41–0.81; *p* < 0.01) (Fig. 3B). Moreover, PFS in the Gr 3–4 group was better than that in the Gr 0–2 group at cutoff C (HR 0.77; 95% CI 0.53–1.13; *p* = 0.18) (Fig. 3C).

## Discussion

This study assessed the relationship between NFF treatment outcomes and neutropenia in patients with pancreatic cancer. Our results showed that neutropenia induced by NFF serves as a useful prognostic factor for pancreatic cancer treatment. To the best of our knowledge, this is the first report to show a relationship between the use of NFF and neutropenia.

On analyzing the relationship between different cutoffs and tumor responses, we found a significant difference at cutoff C. Hamauchi et al. reported that the relationship between the efficacy of TAS-102 in colorectal cancer and neutropenia was verified for multiple cutoffs^13^. This study also used multiple cutoffs to determine whether high grades of neutropenia can be a definitive biomarker of treatment efficacy. Similarly, severe neutropenia has been reported to correlate with high therapeutic efficacy in lung cancer and various other solid tumors^14–17^. The intensity of hematologic toxicity has been reported as an indicator of cytotoxic activity, with strong myelosuppression considered a marker of increased blood levels of anticancer drugs^14,17^. In addition, the sensitivity of the lesions targeted by the treatment, as well as organ functions such as renal and hepatic function, and individual metabolic enzymes, may also affect the effectiveness of the treatment^17,20^. It is also known that neutropenia tends to be particularly pronounced in the early stages of treatment^21^. It will be interesting to investigate whether the timing of neutropenia correlates with treatment response timing, although this relationship cannot be verified in this study. Logistic regression analysis further confirmed the hierarchical importance of cutoffs C, B, and A in descending order for predicting treatment efficacy, with C demonstrating the highest predictive value. Our findings suggest that cutoffs with high grades of neutropenia may be useful indicators of treatment efficacy.

Cutoffs A and B showed significant differences in OS, supported by the adjusted OS data. Therefore, NFF-induced neutropenia could serve as a prognostic factor for patients treated with NFF. However, cutoff C did not show a significant difference in OS, suggesting that while severe neutropenia indicates strong short-term antitumor effects, it might not necessarily correlate with enhanced long-term outcomes.

This study showed a lower rate of Gr 3/4 neutropenia compared to previous reports^22,23^. This may be attributed to the fact that 104 out of 161 patients (64.6%) initially received a reduced dose of 5-FU and/or nalIRI at the start of NFF. Japanese granulocyte colony stimulating factor (G-CSF) guidelines do not typically recommend primary prophylaxis with G-CSF for chemotherapy in solid tumors that are not intended for curative treatment^24^. Patients in the NAPOLEON-2 study were treated according to the Japanese G-CSF guidelines; however, data on the use of G-CSF were not collected.

Fifty-three of 161 patients (33%) received post-treatment in this study. This may have contributed to an OS of 8–9 months, which is better than previous studies, despite a PFS of approximately 3 months. Our literature review revealed no previously reported post-treatment transition rates for NFF. However, it is plausible that the transition rate observed in this study exceeded those of previous investigations. In addition, the prognosis of pancreatic cancer may be determined by the effective management of infections, including cholangitis. It is conceivable that infection control measures in this study were particularly efficacious, potentially contributing to the observed outcomes.

This study also examined the relationship between neutropenia and the RDI of 5-FU and nal-IRI. The results indicated that the group with severe neutropenia had a significantly lower RDI for 5-FU than the group with non-severe neutropenia. Although there was no significant difference in the nal-IRI RDI, the trend was lower in the group with strong neutropenia. These findings suggest that NFF might have a stronger antitumor effect or tendency in patients with severe neutropenia, indicating that the impact of nal-IRI is more substantial than that of 5-FU.

Several inflammatory markers, including CRP, have been studied in the context of cancer. CRP is primarily synthesized in response to inflammatory cytokines, notably interleukin 6^25^. Elevated levels of CRP are commonly observed in patients with cancer. Moreover, elevated WBC counts and CRP levels are established prognostic indicators in solid tumors^26–28^.

Previous studies have demonstrated a relationship between CRP levels and the effectiveness of IRI-based regimens for advanced pancreatic cancer treatment. For instance, among patients who received 5-FU/LV/oxaliplatin after the failure of a gemcitabine-based regimen followed by NFF, those with CRP levels > 1 exhibited poor PFS and OS. Therefore, previous treatments, including nal-IRI, might have impacted the outcomes^29^. Additionally, studies of older patients with pancreatic cancer have reported that those with a low CRP/albumin ratio exhibit good treatment responses^30^. However, in a multivariate analysis where CRP was included as a variable, the group with severe neutropenia showed a significantly better or more favorable trend compared to the group with non-severe neutropenia. This suggest that neutropenia contributes to the therapeutic effect independently of CRP.

However, this study has some limitations that should be acknowledged. First, it may have had some biases due to the retrospective nature of the analysis. Additionally, this study focused only on the Japanese population, and the side effects of IRI may vary among different ethnicities^31,32^. Another limitation was the relatively small number of cases of Gr 3 or 4 neutropenia, especially Gr 4 neutropenia. This may have influenced the lack of significant differences in OS at cutoff C. Clinically, it is important that the group with a higher grade of neutropenia shows superior survival compared to the group with a lower grade of neutropenia in the survival curve at each cutoff. There was no complete response or partial response was observed in cases of *UGT1A1* homozygotes or double heterozygotes, possibly due to the reduced dose of nal-IRI, and only one case of stable disease for > 6 months was reported. However, the number of *UGT1A1* homozygotes and double heterozygotes was difficult to determine in this study because of the small sample size. The timing of neutrophil recovery remains unclear in this study, as the most severe grades of neutropenia were selected, and the study was not initially designed to investigate neutropenia timing. However, the potential for a positive correlation between neutrophil recovery and antitumor efficacy was noted. It is known that prolonged neutropenia increases the risk of febrile neutropenia, which can sometimes be life-threatening^33^. This study did not record the duration of neutropenia; therefore, validating this aspect remains a subject for future research. Further studies should examine the relationship between the timing of neutropenia and tumor response. In addition, significant differences in WBCs, neutrophils, and lymphocytes were observed at the start of treatment at each cutoff. Neutrophils and lymphocytes are both types of leukocytes and can be interconnected. Inflammatory markers calculated from WBC counts and their fractions, albumin levels, and CRP levels at the start of treatment are reported to be biomarkers of OS^28^. Since WBCs and their fractions were designated as inflammatory markers solely in the prognostic index, the cutoff was set at WBC $\:\ge\:$ 11,000. Therefore, the results of the Fisher test for the percentage of WBCs $\:\ge\:$ 11,000 were included in Table 1. There was no significant difference in the proportion of patients with WBC levels above 11,000 at any cutoff. However, since we cannot exclude the possibility that higher baseline WBC and neutrophil counts may be less likely to cause myelosuppression, we aim to reexamine this possibility in the prospective cohort of the NAPOLEON-2 study.

Furthermore, investigating cases with *UGT1A1* homozygous or double heterozygous polymorphisms associated with neutropenia^22,34^ might provide valuable insights into the relationship between neutropenia and antitumor efficacy. Such investigations may help guide treatment decisions, such as considering dose adjustments for nal-IRI in patients with specific *UGT1A1* genotypes, while maintaining safety. There are also reports on dose setting^35–37^, including initial dose reduction and dose increases during treatment, which are not limited to *UGT1A1*, and further validation studies are needed.

To overcome these limitations, future studies should aim to increase the sample size and conduct multinational validations to account for potential ethnic differences in responses and AEs. An ongoing analysis is evaluating whether neutropenia can serve as a biomarker of efficacy in an independent patient population of similar size, aiming to reproduce findings in a prospective cohort analysis of the NAPOLEON-2 study. Building on the results of the NAPOLI-3 study^38^, which validated nal-IRI, 5-FU/LV, and oxaliplatin (NALIRIFOX) as an ongoing first-line treatment, further investigation into the association between neutropenia and the efficacy of NALIRIFOX may be warranted.

In conclusion, NFF-induced neutropenia may serve as a useful prognostic factor for pancreatic cancer patients. It is particularly influenced by nal-IRI and can be considered a novel biomarker.

## Methods

### Study design and patients

This was a retrospective analysis of data from the NAPOLEON-2 study^39^. The NAPOLEON-2 study was a multicenter observational study of pancreatic cancer patients who received NFF therapy at 20 participating institutions in Japan between June 1, 2020, and May 31, 2021, and was tracked until November 30, 2022. No special techniques were applied to eliminate potential bias. There were no additional criteria for this analysis. Based on the results of a survey of participating centers prior to the start of the NAPOLEON-2 study, it was expected that over 100 cases would be accumulated.

### Outcomes

The primary endpoint was overall survival (OS), and the secondary endpoints were the overall response rate, disease control rate, progression-free survival (PFS), adverse events (AEs), and relative dose intensity (RDI). OS was defined as the time from the first NFF administration to patient mortality. PFS was defined as the period from the initiation of NFF administration to the earliest occurrence of disease progression or mortality from any cause. Cases of loss to follow-up were treated as censored observations in OS and PFS analyses. Progression or recurrence was evaluated through regular examinations using the Response Evaluation Criteria in Solid Tumors (RECIST) version 1.1^40^. The response rating was the best rating.

Since this was not a prospective study, it was difficult to standardize the timing of treatment evaluation. The timing of CT evaluations varied among institutions, attending physicians, and as the patient’s medical condition changed. The timing of neutropenia assessment and diagnostic imaging was not always synchronized. Whether blood tests were scheduled and computed tomography (CT) were performed on the same day depended on the facility and the attending physician. Since NFF is administered bi-weekly, blood tests were performed accordingly, minimizing significant discrepancies between the dates of neutropenia and CT evaluations. As per the results of the NAPOLI-1 study and the Japanese phase 2 study^4–6^, nal-IRI 70 mg/m^2^, 5-FU 2400 mg/m^2^, and LV 200 mg/m^2^ given every 2 weeks were used as the reference doses. At the start of NFF, reduced doses of 5-FU and/or nal-IRI were administered to 104 out of 161 patients (64.6%). Initial dose reduction and dose modification during treatment were permitted at the physician’s discretion^39^.

### Statistical analysis

Neutropenia was assessed according to the Common Terminology Criteria for Adverse Events version 5.0^41^. Neutropenia was assessed at the lowest grade [Gr] throughout the entire NFF treatment period. We used three cutoff points for classification: cutoff A (Gr 0 versus Gr 1–4), cutoff B (Gr 0–1 versus Gr 2–4), and cutoff C (Gr 0–2 versus Gr 3–4). Fisher’s exact test and Mann–Whitney’s U test were used to compare group differences by their respective cutoff values. The relationship between each cutoff and tumor response was assessed using RECIST version 1.1. Continuous data, such as blood test results at the start of treatment and RDI, were compared using the Mann–Whitney U test. Categorical data, such as sex and performance status (PS), were compared using the Fisher’s exact test. Two-sided Fisher’s exact and odds ratio tests were conducted to assess associations between treatment response (complete response, partial response, or no response according to RECIST version 1.1) and neutropenia grade (high or low) at each cutoff. In addition, logistic regression analysis was performed with age (≥ 75 years/<75 years), initial dose (yes/no), number of prior chemotherapies (1/≥2), Eastern Cooperative Oncology Group PS (0/≥1), the sum of target lesions (≥ 50 mm/<50 mm), and cutoff included as variables.

OS and PFS were estimated using the Kaplan–Meier method and compared by the log-rank test. Hazard ratios (HRs) were estimated by Cox proportional hazard regression analysis. The analysis was performed using a 95% CI. Differences were considered statistically significant if the p-value was < 0.05. In addition, univariate and multivariate Cox regression analyses were performed for OS, considering variables such as age (≥ 75 years/<75 years), PS (0/≥1), number of prior chemotherapies (1/≥2), presence of ascites (yes/no), CA19-9 levels (≥ 1000/<1000), C-reactive protein (CRP) levels (≥ 1/<1), and each cutoff. These covariates were selected by clinicians based on the evaluation of significant variables reported in previous literature and actual patient backgrounds prior to looking at the results^42^. These were selected as clinically meaningful variables with p-values < 0.05. Missing values were not complemented.

All statistical analyses were performed using EZR (Saitama Medical Center, Jichi Medical University, Saitama, Japan), which is a graphical user interface for R (The R Foundation for Statistical Computing, Vienna, Austria). More precisely, it is a modified version of the R commander designed to add statistical functions frequently used in biostatistics^43^.

## Electronic supplementary material

Below is the link to the electronic supplementary material.

Supplementary Material 1

## Data Availability

All data generated or analyzed in this study are stored in a secured research database. Although they are not publicly available, they are available through the corresponding author upon reasonable request.
