# Supplementary material for: Relationship between neutropenia caused by nanoliposomal irinotecan/fluorouracil/leucovorin and treatment outcomes in the NAPOLEON-2 study (NN-2301)
Source: Sci Rep. 2025 Jan 27;15:3427. doi: 10.1038/s41598-025-88005-4 (PMC11772893; doi:10.1038/s41598-025-88005-4)
Supplement: Supplementary file 1 — Supplementary Material 1 [file 41598_2025_88005_MOESM1_ESM.docx]

**Supplementary Table 1. Sensitivity Analysis in Cox Regression**

|  |  | **HR** | **95% CI** | **p-value** |
| --- | --- | --- | --- | --- |
| Age |  | 0.99 | 0.96–1.01 | 0.22 |
| Sex | Male | 1.13 | 0.77–1.66 | 0.54 |
| CRP |  | 1.14 | 1.08–1.21 | p < 0.01 |
| Log CA19-9 |  | 1.17 | 0.99–1.38 | 0.07 |
| Cutoff A | Gr 1-4 | 0.72 | 0.49v1.07 | 0.11 |

|  |  | HR | 95% CI | p-value |
| --- | --- | --- | --- | --- |
| Age |  | 0.99 | 0.96–1.01 | 0.22 |
| Sex | Male | 1.13 | 0.77–1.66 | 0.55 |
| CRP |  | 1.14 | 1.08–1.21 | p < 0.01 |
| Log CA19-9 |  | 1.17 | 0.99–1.38 | 0.07 |
| Cutoff B | Gr 2-4 | 0.71 | 0.48–1.06 | 0.09 |

|  |  | HR | 95% CI | p-value |
| --- | --- | --- | --- | --- |
| Age |  | 0.99 | 0.96–1.01 | 0.21 |
| Sex | Male | 1.17 | 0.80–1.72 | 0.42 |
| CRP |  | 1.14 | 1.09–1.21 | p < 0.01 |
| Log CA19-9 |  | 1.17 | 0.99–1.39 | 0.07 |
| Cutoff C | Gr 3-4 | 0.84 | 0.53–1.32 | 0.44 |
